# Infective Endocarditis With Secondary Headache: A Case Report

**DOI:** 10.7759/cureus.26791

**Published:** 2022-07-12

**Authors:** Keita Takizawa, Kana Ozasa, Kunihito Matsumoto, Jumi Nakata, Noboru Noma

**Affiliations:** 1 Department of Oral Medicine, Nihon University School of Dentistry, Tokyo, JPN; 2 Division of Internal Medicine, Towa Hospital, Tokyo, JPN

**Keywords:** streptococcus viridans, fever, headache, apical periodontitis, infective endocarditis

## Abstract

Secondary headache is a symptom of an underlying disease. Infective endocarditis (IE) is a serious infection of the heart tissue. Herein, we present a rare case of IE, with a secondary headache. The patient presented with persistent headache, fever of 39°C, myalgia, and painful erythema of the plantar surface of the foot. The headache progressively worsened over a few weeks. She was diagnosed with secondary headache, and sepsis was suspected. Blood culture revealed the presence of *Streptococcus viridans,* leading to a diagnosis of IE. Postoperatively, the patient recuperated without any complications. Headaches can be secondary to other conditions. Therefore, comprehensive assessment and accurate diagnosis are essential. Dentists must be aware that headache is a concomitant symptom of IE.

## Introduction

Secondary headache, also known as organic headache, is a symptom of an underlying disease or condition. A combination of headache and fever prompts the clinician to rule out systemic/neurological infections, vasculitis, rheumatic disease, or other inflammatory diseases [1]. Neurological infections include bacterial meningitis, viral meningitis, encephalitis, and brain abscesses. In the presence of systemic infections, a headache is usually a relatively inconspicuous symptom and diagnostically unhelpful. Systemic infections are usually dominated by fever, general malaise, and other systemic symptoms. Nevertheless, some systemic infections, particularly influenza, are accompanied by headache, as a prominent symptom, along with fever [2].

Infective endocarditis (IE) is characterised by inflammation of the valves, endocardium, and macrovascular intima. This can cause bacteraemia, vascular embolism, and heart damage. It is a systemic septic disease with clinical symptoms [3]. The incidence of IE is low. However, it can lead to multiple complications, including death, if not diagnosed promptly and treated appropriately [3,4]. Caries and periodontal disease are common oral diseases, caused by indigenous bacteria in the oral cavity. Indigenous oral bacteria may invade blood vessels during surgical procedures, such as tooth extraction and removal of tartar, causing bacteraemia [5]. However, poor oral hygiene is more likely to cause bacteraemia. It is necessary to exercise caution if the patient has caries or periodontal disease and is susceptible to bacteraemia. Therefore, dentists must understand the role of dental diseases in the development of IE and secondary headaches [6]. Here, we describe a rare case of IE with secondary headache.

## Case presentation

**Figure 1:**
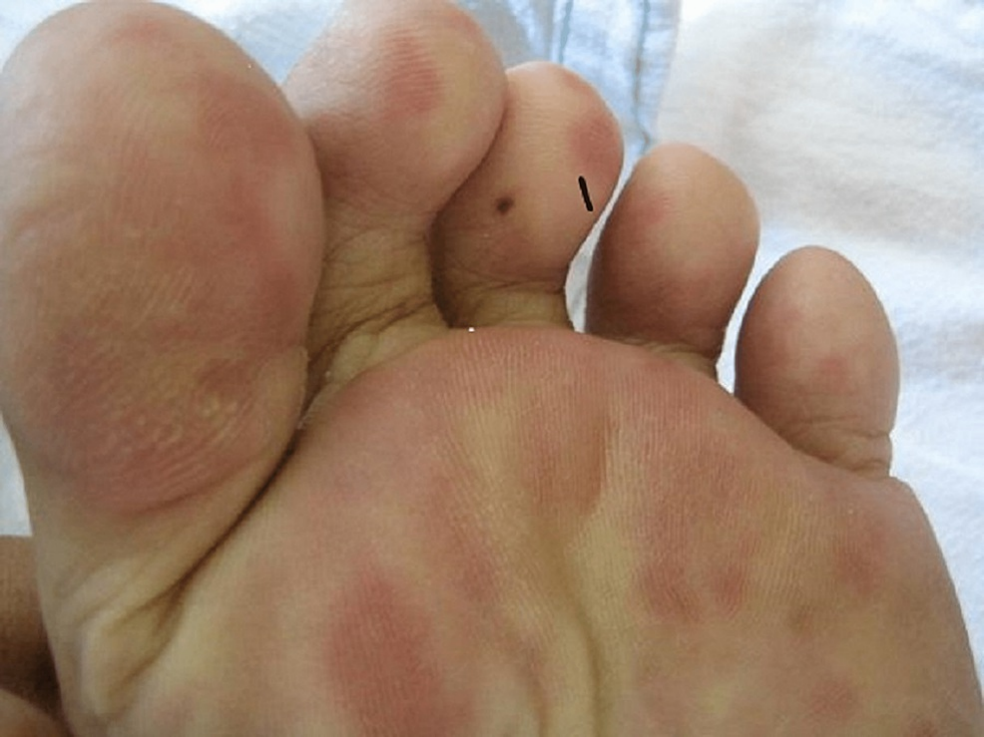
Painful erythema on the plantar surface of the foot

The patient was a 51-year-old female who visited the outpatient clinic at our hospital for a headache. The patient had been experiencing general fatigue for two weeks and, from two days before visiting our hospital, became aware of insect bites on her right sole. Subsequently, she developed a headache, fever of 39°C, weight loss, and myalgia. The fever persisted. Her laboratory data showed high inflammatory reaction; therefore, she was hospitalised the next day for detailed examination and treatment. Her vital signs at the time of consultation were as follows: consciousness level, clear; body temperature, 39.4°C; blood pressure, 122/73 mmHg; pulse rate, 113/min; and oxygen saturation (SpO_2_), 99%. Her headache progressively worsened with the fever and spread to the whole head. The headache was persistent and moderate to severe in intensity. Systemic findings were as follows: painful erythema on the sole of the right foot (multiple Osler's nodes), generalised myalgia, reduced dexterity of the upper limbs, weakness of the lower limbs, and gait disturbance. The facial features were symmetrical, and no trismus was observed (Figure 1).

The following oral findings were observed: the right maxillary second molar, left maxillary first molar, and right mandibular first molar were residual roots with plaque accumulation. In addition, swelling and redness were observed in the gingiva surrounding the residual roots. Blood tests performed at the first visit showed high inflammatory reaction. High levels of aspartate aminotransferase (AST), alanine aminotransferase (ALT), lactate dehydrogenase (LDH), alkaline phosphatase (ALP), γ-glutamyl transpeptidase (γ-GTP), total bilirubin (TB), and creatine kinase (CK) were observed, indicating liver dysfunction and muscle disorder. In addition, her procalcitonin (PCT) level was high, indicating the presence of sepsis (Table 1).

**Table 1:** The results of blood tests at the first visit PLT: platelet, HGB: haemoglobin, AST: aspartate aminotransferase, ALT: alanine aminotransferase, LD: lactate dehydrogenase, ALP: alkaline phosphatase, γ-GTP: γ-glutamyl transpeptidase, T-Bil: total bilirubin, CK: creatine kinase, BUN: blood urea nitrogen, CRE: creatinine, PCT: procalcitonin, CRP: C-reactive protein.

Laboratory parameters with units	Patient values	Reference range
RBC (10^4^/μL)	412	353-466
WBC (10^3^/μL)	7.1	3.0-7.8
PLT (10^4^/μL)	12.8	13.8-30.9
HGB (g/dL)	6.9	10.6-14.4
AST (IU/L)	108	≦ 30
ALT (IU/L)	117	≦ 30
LD (IU/L)	328	119-229
ALP (IU/L)	402	38-113
γ-GTP (IU/L)	142	≦ 50
T-Bil (mg/dL)	1.4	0.2-1.2
CK (IU/L)	387	45-163
BUN (mg/dL)	11.1	7-24
CRE (mg/dL)	0.72	≦ 0.70
PCT (ng/mL)	19.6	≦ 0.05
CRP (mg/dL)	20.4	≦ 0.3

**Figure 2:**
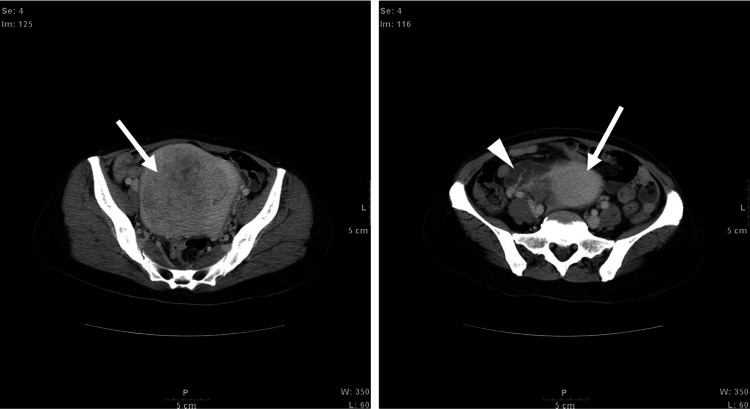
Contrast-enhanced computed tomography (CT) of the thorax and abdomen Findings in the lower abdomen are suggestive of uterine fibroids and tubal cystic mass. Chest and abdominal contrast-enhanced computed tomography (CT) scans showed high-density lesion (uterine fibroid) in the middle (arrow) of the lower abdomen and a superposed tubular structure with fluid collection in the area corresponding to the right fallopian tube (arrowhead) in contact with it (suspected ovarian cyst). P: posterior, W: window width, L: window level, Se: series number, Im: image number.

Blood tests revealed systemic organ damage, high inflammatory response, and high levels of PCT. Therefore, sepsis was suspected. Various diagnostic imaging modalities were used to identify the causative organism. The causative bacteria were identified using blood culture. Chest CT and radiographs showed no abnormal findings in the lung field or the longitudinal sac and no cardiac enlargement. No obvious abnormal findings were found on electrocardiography. Thoracoabdominal CT showed suspicious uterine fibroids and tubal cysts in the lower abdomen (Figure 2).

**Figure 3:**
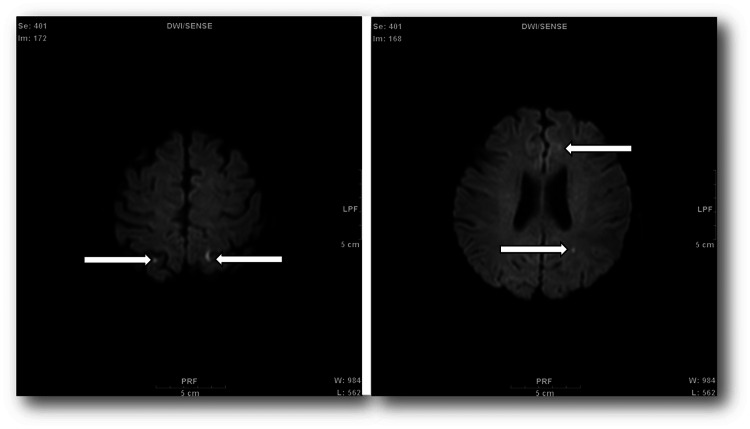
Magnetic resonance imaging (MRI) of the head Multiple cerebral infarcts can be observed with no indication of the source of infection. Magnetic resonance imaging (MRI) (horizontal diffusion-weighted image) of the head revealed multiple small hyperintensities (arrows) (suspected lacunar infarction). W: window width, L: window level, Se: series number, Im: image number, LPF: left posterior foot, PRF: posterior right foot, DWI: diffusion-weighted image, SENSE: sensitivity encoding.

No subjective abdominal symptoms or pain would have been observed during the abdominal examination if the oviductal cystoma was the source of infection. In addition, muscle weakness and gait disorder would not have been observed. Head MRI was performed because of suspected infection (Figure 3). Magnetic resonance imaging (MRI) (horizontal diffusion-weighted image) of the head revealed multiple small hyperintensities (arrows) (suspected lacunar infarction). No widespread infarct or infection focus was observed.

**Figure 4:**
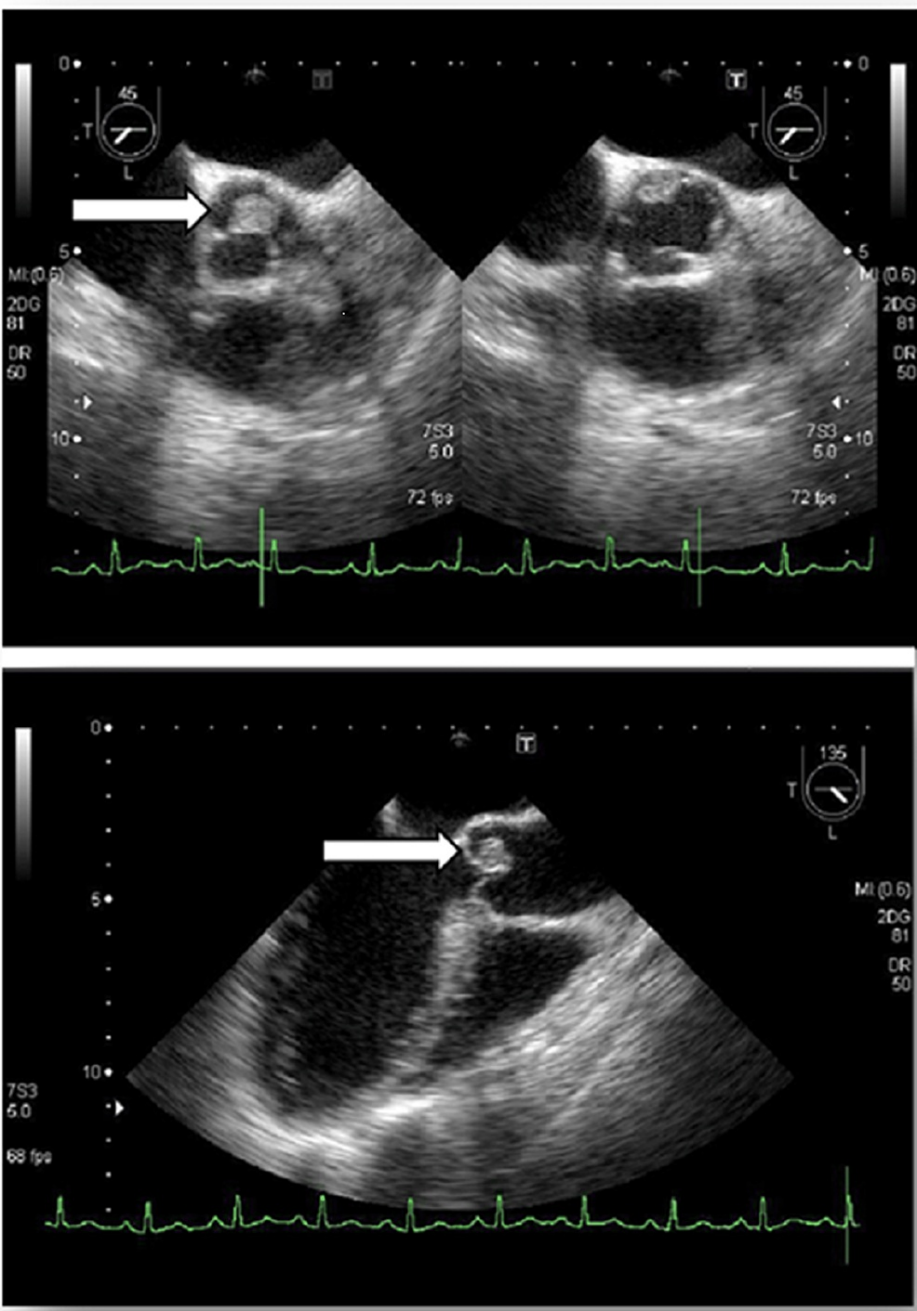
Echocardiographic image of 9×10 mm verrucae on the aortic valve Echocardiography (at the first visit) showed a 9×10-mm-sized floating hyperechoic mass (arrow) attached to the aortic valve (suspected vegetation formation). T: transverse plane, L: left atrium, MI: mechanical index, DR: dynamic range.

However, although multiple cerebral infarctions were found in the brain, there were no findings suggestive of the presence of infection. Subsequently, her blood culture showed *Streptococcus viridans*, an indigenous bacterium of the oral cavity. Echocardiographic images showed warts measuring 9×10 mm on the aortic valve; no abnormality was found on electrocardiography (Figure 4).

**Figure 5:**
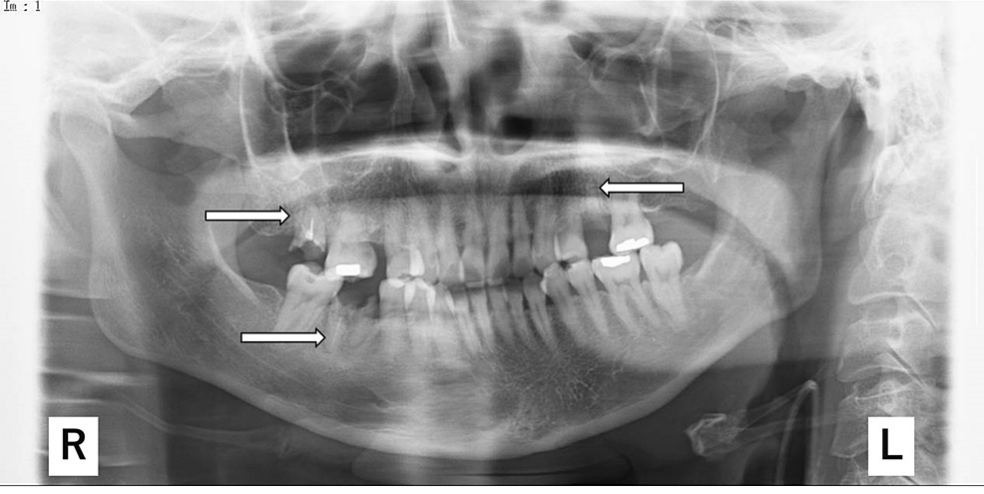
Panoramic radiographs Panoramic radiography revealed that images 17, 26, and 46 (arrows) show residual roots and radiolucent images of the right maxillary second molar, left maxillary first molar, and right mandibular first molar, respectively. X-ray transmission revealed osteosclerosis around the apex of the right maxillary second molar and the right mandibular first molar (chronic apical periodontitis). Im: image number, R: right, L: left.

Panoramic radiography scans showed the residual roots of the right maxillary second molar, left maxillary first molar, and right mandibular first molar. The X-ray transmission images revealed osteosclerosis around the apex of the right maxillary second molar and the right mandibular first molar (Figure 5).

**Figure 6:**
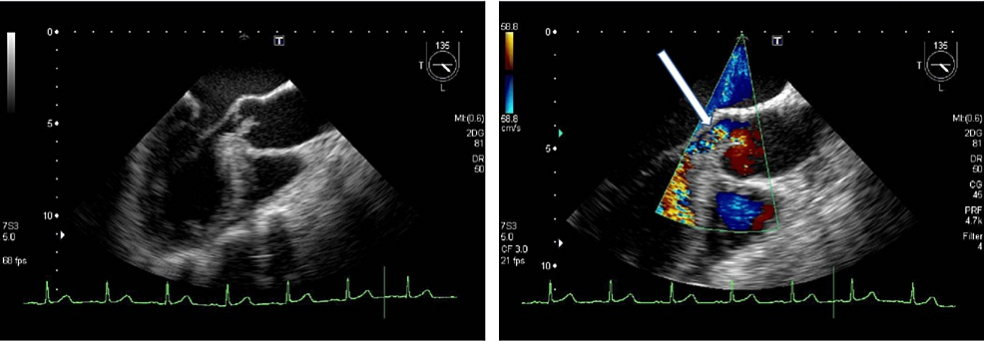
Echocardiographic image Aortic valve perforation (arrow) can be visualised. Im: image number, T: transverse plane, L: left atrium, CG: colour gain, PRF: pulse repetition frequency, CF: colour frequency, DR: dynamic range, 2DG: two-dimensional gain, MI: mechanical index.

Based on these findings, the patient was diagnosed with IE and was started on an infusion of penicillin G at 24 million units/day and gentamicin at 150 mg/day. The blood culture performed three days later showed negative findings. The blood test findings improved; however, auscultation revealed a heart murmur. Echocardiography was performed again, and perforation of the aortic valve was observed (Figure 6).

Considering the risk of reinfection with the artificial valve, we extracted the three residual root teeth, which were the sources of infection and provided guidance on oral hygiene management before performing aortic valve replacement. Aortic valve replacement was performed on June 8 of the same year. Motor dysfunction persisted because of the cerebral infarction caused by warts, but the patient’s clinical course remains satisfactory.

## Discussion

In the International Classification of Headache Disorders, Third Edition, “Headache attributed to systemic infection (9.2)” is divided into three categories: headache attributed to systemic bacterial infection (9.2.1), headache attributed to systemic viral infection (9.2.2), and headache attributed to other systemic infection (9.2.3) [2]. However, some systemic infections, especially influenza, have headache and fever as prominent symptoms. If systemic infection is accompanied by meningitis or encephalitis, the headache occurring in that situation should be encoded under the 9.1 headache category, i.e., headache caused by intracranial infection. In the presence of infections, headache usually co-occurs with fever, but headache can also occur in the absence of fever. The mnemonic SNOOP (systemic symptoms/signs and disease, neurologic symptoms or signs, onset sudden or onset after the age of 40 years, and change of headache pattern) was proposed to help clinicians detect red flags for secondary headaches [7].

Systemic diseases include fever, weight loss, chills, myalgia, and anorexia. Systemic signs, including headache, may occur secondary to medical conditions such as acquired immunodeficiency syndrome, cancer, vasculitides, septic or aseptic meningitis, early encephalitis, and cerebrovascular disorders [7]. In the case of our patient, the differential diagnoses included giant cell arteritis, meningitis, and systemic infection. Early symptoms of giant cell arteritis are related to systemic inflammation and include fatigue, malaise, weight loss, fever, night sweats, anorexia, and weakness.

Patients with giant cell arteritis experience allodynia due to tender temporal arteries or nodules, which may develop on the scalp, especially on areas over inflamed arteries, and intermittent claudication, a gradual feeling of discomfort, weakness, and tension after the use of the masseter and temporalis muscles (jaw claudication) [8,9]. The gold standard for confirming giant cell arteritis is a biopsy of the temporal artery [10,11]. Foreign giant cells may be visualised on histological exam, and blood tests may show elevated erythrocyte sedimentation rate (ESR) and C-reactive protein (CRP) levels [12]. Our patient’s clinical signs and symptoms indicated giant cell arteritis, but she did not have allodynia, jaw claudication, and diplopia. Furthermore, her blood test showed an elevated CRP level (20.4 mg/dL). On the other hand, meningitis is a typically acute condition, associated with neck stiffness, nausea, fever, and changes in mental state. For the above reasons, we were able to exclude both arteritis and meningitis because the patient did not show the signs and symptoms and her blood culture yielded negative results.

Additionally, the patient had cerebral infarction, but the headache was not due to ischemic cerebral infarction. Headaches due to ischemic cerebral infarction may be accompanied by paralysis of limbs and cranial nerve symptoms. In cases of cerebral infarction in IE, such as this case, it is necessary to consider haemorrhagic infarction. IE is a systemic septic disease that involves vegetation formation including bacterial clusters in the valves, endocardium, and large intima. Patients with IE present with various clinical symptoms such as bacteraemia, vascular embolism, and cardiac disorders [13].

The modified Duke diagnostic criteria are used to confirm IE; however, in this case, cardiac ultrasonography revealed a 9×10-mm swaying lesion in the aortic valve in addition to vascular phenomena such as major vascular embolisation, fever, and Osler's node. A certain immune phenomenon, gram-positive bulbous bacterium, was detected in two sets of blood cultures, and microbiological findings, such as high active inflammatory response and PCT positivity, were observed. Therefore, IE was diagnosed since two major and three minor criteria were satisfied [14].

This patient was not diagnosed with IE at an early stage, since it took three weeks from the appearance of the initial symptom of general malaise to the definitive diagnosis in early May. Early diagnosis of IE is difficult since the clinical signs of fever of ≥38°C and new heart murmurs are frequent findings; however, other findings are infrequent and lack characteristic features [15]. In the current case, the patient had a fever of 39°C for more than two weeks, in addition to myalgia and headache; she also had a heart murmur (systolic murmur at the left sternal border (2LSB)). Furthermore, the diagnosis was delayed due to nonspecific physical symptoms such as painful erythema on the sole of the foot, reduced dexterity of the upper limbs, muscle weakness of the lower limbs, and gait disturbance. If fever and heart murmur are present for a long period of time (e.g., more than three weeks), IE should be suspected, and blood culture and echocardiography should be performed.

According to the IE statistics in Japan, *S. viridans* is the most common causative bacteria (56.7%), and “tooth extraction and other dental treatment” are the most common causes (45.7%). This means that *S. viridans* enter the bloodstream through the oral cavity [16,17]. However, according to Wilson et al., most patients with IE have no history of dental treatment such as tooth extraction. It takes approximately two weeks for the symptoms to appear if a tooth extraction is the cause of IE, and in a previous study, 7% of the patients underwent dental treatment 7-14 days before the incubation period [18]. In this case, *S. viridans*, an indigenous bacterium in the oral cavity, was detected in the blood culture, and apical periodontitis was observed to have spread to the jawbone. Hence, IE could be attributed to the spread of *S. viridans* infection to the jawbone and the decrease in immune function associated with hyponutrition. At the onset of IE, three teeth with apical periodontitis remained, and X-ray transmission images revealed osteosclerosis around the apex of the right maxillary second molar and the right mandibular first molar. Due to the lack of subjective symptoms and the possibility that the apical periodontitis was inactive, treatment opportunities were missed, and *S. viridans* proliferated in the alveolar bone. It was presumed that *S. viridans* had spread in her body.

In this case, anaemia developed as a concomitant symptom of IE. Prolonged systemic inflammation for more than a month results in a shortened erythrocyte lifespan due to inflammatory cytokines, decreased erythropoietin production, decreased iron utilisation, and ferritin accumulation in the liver. In this case, anaemia may have developed due to the prolonged inflammation. In patients with abscess formation, the range of inflammation due to IE is wide, and the prognosis is poor without surgical treatment; therefore, emergency surgery is indicated in such cases. After artificial valve replacement surgery, inflammation of the sewn section of the artificial valve may cause perivalvular regurgitation from the fragile part [3]. If the range of inflammation is expanded, a high degree of regurgitation can occur, resulting in excessive movement of the artificial valve. This is an extremely dangerous sign of valve dysfunction [3]. In this case, the blood culture performed three days after the administration of antibiotic medication showed negative culture findings, and the inflammatory findings had improved; however, echocardiography revealed perforation of the aortic valve, and surgery was considered. Owing to the risk of reinfection after artificial valve replacement, we advised the patient to undergo extraction of the causative teeth and provided guidance on oral hygiene management before performing aortic valve replacement.

## Conclusions

In patients scheduled for aortic valve replacement surgery, it is important to maintain proper oral hygiene and to extract diseased teeth prior to surgery. In the hyperacute phase of IE, the priority must be to prevent death, so ideally all teeth serving as possible sources of infection should be extracted and aortic valve replacement should be performed. It is the role of the dentist to provide comprehensive periodontal therapy and routine dental cleaning once the acute phase of IE has passed. Dentists must be aware of and look for red flags during comprehensive assessments for secondary headaches.
